# mRNA Levels of Aromatase, 5α-Reductase Isozymes, and Prostate Cancer-Related Genes in Plucked Hair from Young Men with Androgenic Alopecia

**DOI:** 10.3390/ijms242417461

**Published:** 2023-12-14

**Authors:** Pilar Sánchez, Cristina Serrano Falcón, Sergio Martínez Rodríguez, Jesús M. Torres, Salvio Serrano, Esperanza Ortega

**Affiliations:** 1 Department of Biochemistry, Molecular Biology and Immunology, Faculty of Medicine, University of Granada, 18016 Granada, Spain; pilarsan@ugr.es (P.S.); sergio@ugr.es (S.M.R.); 2 Hospital of Guadix, 18500 Granada, Spain; cristinaserranof23@gmail.com; 3 Department of Dermatology, Faculty of Medicine, University of Granada, 18012 Granada, Spain; salvioserrano2023@gmail.com

**Keywords:** androgenic alopecia, aromatase, 5α-R isozymes, prostate cancer genes, trichogram

## Abstract

Androgenic alopecia (AGA) is the most prevalent type of progressive hair loss and has psychological repercussions. Nevertheless, the effectiveness of current pharmacological treatments remains limited, in part because the molecular basis of the disease has not been fully elucidated. Our group previously highlighted the important roles of aromatase and 5α-reductase (5α-R) in alopecia in young women with female pattern hair loss. Additionally, an association has been proposed between AGA and prostate cancer (PCa), suggesting that genes implicated in PCa would also be involved in AGA. A low-invasive, sensitive, and precise method was used to determine mRNA levels of aromatase, 5α-R isozymes, and 84 PCa-related genes in samples of plucked hair from young men with AGA and controls. Samples were obtained with a trichogram from the vertex scalp, and mRNA levels were quantified using real-time RT-PCR. The men with AGA had significantly higher 5α-R2 mRNA levels in comparison to controls; interestingly, some of them also showed markedly elevated mRNA levels of 5α-R1 or 5α-R3 or of both, which may explain the varied response to 5α-R inhibitor treatments. The men with AGA also showed significant changes versus controls in 6 out of the 84 genes implicated in PCa. This study contributes greater knowledge of the molecular bases of AGA, facilitating early selection of the most appropriate pharmacological therapy and opening the way to novel treatments.

## 1. Introduction

Androgenic alopecia (AGA), also known as male pattern baldness, is a common dermatologic condition characterized by progressive miniaturization of the hair follicle (HF), leading to vellus transformation of terminal hair [1]. In males, AGA courses with recession of the frontal hairline, hair thinning over the vertex scalp, and balding [2]. The severity and age of onset of hair loss vary; it usually affects older men but can be observed from the age of 30 years [3]. The psychological consequences of this disease can be damaging in a society that places a high value on hair, which is associated with attractiveness [4].

Genetic and hormonal factors and the presence of systemic diseases have been implicated in the etiology of AGA, although its precise cause has yet to be elucidated [5]. However, the link between AGA and androgens is well established, and the progression of AGA is known to require dihydrotestosterone (DHT), which is the active metabolite of testosterone (T) and possesses a five-fold greater affinity for androgen receptors (ARs) than T, facilitating its binding to ARs [6]. The binding of DHT to ARs in HFs leads to miniaturization of the follicle by shortening the duration of the anagen phase of hair growth and prolonging its telogen (resting) phase, producing an increase in immature hair and a decrease in new hair [7,8]. A balding scalp is characterized by high DHT levels and increased AR expression [9].

DHT is synthesized from T by the steroid 5α-reductase (5α-R) enzyme. There are three well-characterized 5α-R isozymes in the HF: type 1 (SRD5A1), type 2 (SRD5A2), and type 3 (SRD5A3) [10]. The 5α-R2 isozyme plays a key role in the etiology of AGA, and an inhibitor of this isozyme, finasteride, is used in its treatment [11]. Furthermore, patients with male pseudohermaphroditism do not suffer from AGA because they lack 5α-R2, suggesting that DHT is essential for hair growth [12].

Aromatase (CYP19A1), which is required to convert androgens to estrogens, has been detected in scalp HFs [13] and may also play a major role in AGA, as previously reported by our group in women with female pattern hair loss (FPHL) [10].

Androgens and ARs have been found to participate in the development, progression, and metastasis of prostate cancer (PCa) [14,15], and there is growing evidence that estrogens might assist androgens in perpetuating carcinogenesis [16]. Given that AGA and PCa appear to share some common pathophysiological mechanisms, including age, hereditability, and endogenous hormones [17,18], various authors have investigated a possible link between AGA and PCa. Some suggested that AGA onset, especially in younger males (under 30 years) with hair loss involving the vertex, might be a marker of increased PCa risk later in life [19,20,21,22,23,24], whereas others found no association between AGA and an increased risk of PCa [25,26,27]. These discrepancies may be attributable to differences in study design. If a relationship between PCa and AGA exists, some genes implicated in PCa might also be involved in AGA.

With this background, the objective of this study was to explore the molecular bases involved in AGA in young men in order to support the selection of the most appropriate pharmacological therapy and lay the groundwork for the development of novel treatments in the future. For this purpose, a low-invasive, sensitive, and precise method was applied to quantify mRNA levels of aromatase and 5α-R isozymes, and PCR array technology was used to determine mRNA levels of 84 key genes related to the development of PCa.

## 2. Results

### 2.1. 5α-R Isozymes and Aromatase mRNA Levels

**Figure 1:**
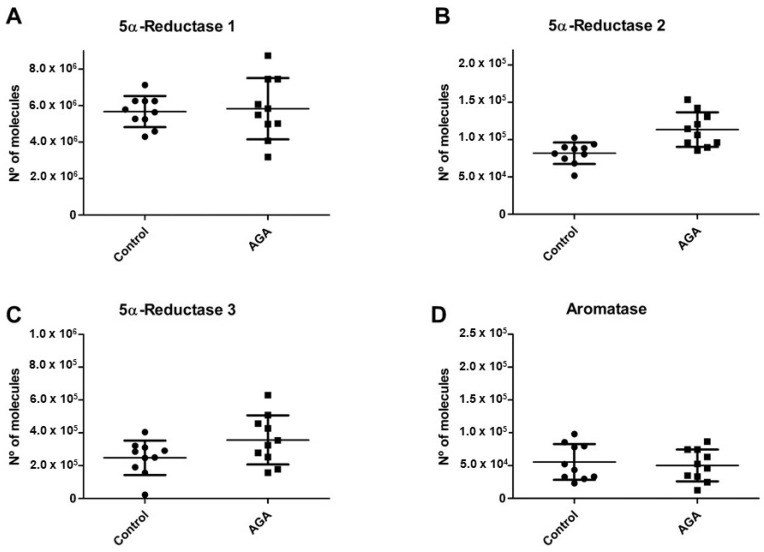
mRNA levels of 5α-Reductase type 1 (5α-R1) (**A**), 5α-Reductase type 2 (5α-R2) (**B**), 5α-Reductase type 3 (5α-R3) (**C**), and aromatase (**D**) in anagen hairs plucked from young men with AGA (*n* = 10) and controls (healthy men with no hair loss or thinning) (*n* = 10). The 5α-R2 mRNA levels were significantly higher in the AGA group than in controls (*p* = 0.0015). Data are expressed as means ± SD.

In the global series of plucked hair samples (from men with AGA and controls), the highest mRNA levels were for 5α-R1, followed by 5α-R3, and then 5α-R2 (Figure 1A–C).

The 5α-R2 mRNA levels were significantly higher in the AGA group versus controls (*p* = 0.0015) (Figure 1B). Higher mean mRNA levels of 5α-R3 isozymes (*p* = 0.1875) (Figure 1C) were also observed in the AGA group, although the difference did not reach statistical significance. The 5α-R1 (*p* = 1.0000) (Figure 1A) and aromatase mRNA levels (*p* = 0.8534) (Figure 1D) did not significantly differ between the AGA and control groups. However, the figures depict a wide variability in results for 5α-R1 and 5α-R3 isozymes among the men with AGA.

### 2.2. Analysis of Gene Expression Profiles via PCR Array

**Figure 2:**
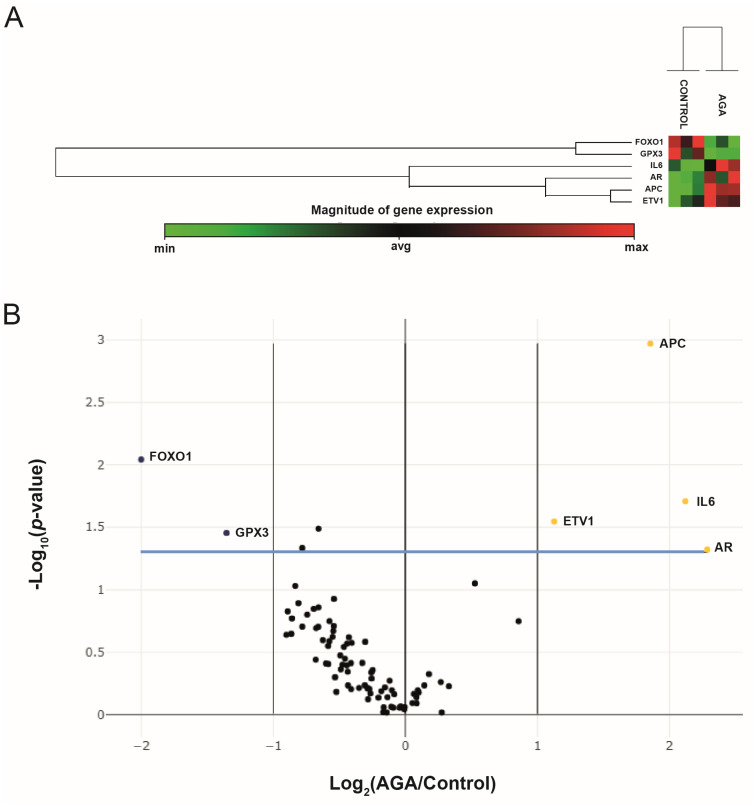
PCR-array analysis showing the quantification of mRNA levels of 84 genes implicated in prostate cancer in plucked hair from young men with AGA and controls. (**A**) Cluster analysis. The color intensity from green to red shows the degree of downregulation (green) to upregulation (red) compared with the other samples. A group of three control cases is clustered on the right side, and a group of three young men with AGA cases is clustered on the left side. (**B**) Volcano plot showing gene expression differences between young men with AGA and control groups by plotting the log_2_ of fold changes in gene expression on the *x*-axis against their statistical significance on the *y*-axis. Each spot represents a single gene. The left vertical axis indicates a two-fold lower expression in young men with AGA versus the control group (blue spots); the central vertical axis shows no change in gene expression; and the right vertical axis indicates a two-fold higher expression in young men with AGA versus controls (yellow spots). The blue line indicates the *t*-test threshold (*p* < 0.05). Statistically significant upregulations/downregulations of genes are indicated by the gene symbols *FOXO1* (Forkhead box O1), *GPX3* (glutathione peroxidase 3 (plasma)), *IL6* (interleukin 6 (interferon, beta 2)), *AR* (androgen receptor), *APC* (adenomatous polyposis coli), and *ETV1* (Ets variant 1). Three independent experiments were performed.

PCa-related gene expressions in the studied hair samples were determined using the Human PCa RT2 Profiler PCR Array. Two housekeeping genes (HKGs) were used for normalization, namely hypoxanthine phosphoribosyltransferase 1 (HPRT1) and ribosomal protein, large, P0 (RPLP0). Among the eighty-four genes in this array, six showed a more than two-fold difference in expression between the AGA and control groups: four genes were upregulated in the AGA group versus the control group (APC (3.62-fold; *p* = 0.001070), AR (4.87-fold; *p* = 0.047702), ETV1 (2.18-fold; *p* = 0.028468), and IL6 (4.35-fold; *p* = 0.019576)), whereas two genes were downregulated in the AGA group versus controls (*FOXO1* (−4.00-fold; *p* = 0.009073) and *GPX3* (−2.56-fold; *p* = 0.035205)) (Table 1 and Figure 2A). These genes are relevant to multiple PCa signaling pathways, including differentially Methylated Promoters (*APC*, *GPX3*), upregulated in PCa (*ETV1*), Androgen Receptor Signaling (*AR*, *IL-6*, *FOXO1*), AKT & PI3 Kinase Signaling (*AR*, *IL-6*, *FOXO1*), PTEN Signaling (IL-6), Apoptosis (*ETV1*, *IL-6*), Cell Cycle (*APC*), and Transcription Factors (*AR*, *ETV1*, *FOXO1*) (Table 2). Overall differences in gene expression patterns for young men with AGA versus controls are represented as a “volcano plot”, in which log 2-transformed fold changes in gene expression are plotted against *p*-values of the Student’s *t*-test; genes plotted further from the central axes showed greater fold changes (Figure 2B).

## 3. Discussion

In this study, 5α-R2 mRNA levels were significantly higher in plucked hair from young men with AGA than from controls, but there was no significant between-group difference in mean 5α-R1 or 5α-R3 mRNA levels. Previous studies based on scalp biopsies observed higher 5α-R1 and 5α-R2 levels in frontal versus occipital HFs from men with AGA [28], while others reported higher 5α-R2 expression in dermal papilla cells (DPCs) from AGA scalps than in DPCs from other sites [29,30]. Importantly, our results were obtained using a low-invasive methodology that does not require a biopsy.

Although there was no difference in mean levels of 5α-R1 or 5α-R3 mRNA with controls, HFs from some of the men with AGA had very high mRNA levels of 5α-R1 or 5α-R3 or both isozymes, which may explain reported variations in the response to 5α-R inhibitor treatments. In this way, although oral finasteride is a well-established treatment for AGA [31], it has been found to offer no hair growth improvement in around 35% to 53% of men [32]. Given the wide variability in mRNA levels of 5α-R isozymes observed in the present study, some men with AGA may be more effectively treated with dutasteride, a 5α-R inhibitor that exerts an inhibitory effect on all three 5α-R isozymes, as reported in the prostate [33]. In fact, two meta-analyses have concluded that oral dutasteride is more effective than oral finasteride to treat male AGA [11,34].

No significant difference in aromatase mRNA was found between the men with AGA and controls, unlike our previous report of a significant difference in women with FPHL [10]. Thus, aromatase activity in hair follicles varies between the sexes [28].

Our present and previous findings on 5α-R isozymes and aromatase suggest that the etiologies of AGA and FPHL differ at the molecular level.

Six of the 84 PCa-related genes significantly differed between the men with AGA and the controls, with *AR*, *IL-6*, *ETV1*, and *APC* being upregulated and *FOX1* and *GPX3* downregulated in comparison to controls.

AR is a member of the nuclear receptor superfamily that functions as a ligand-dependent transcription factor, and *AR* gene overexpression has been described in 30–50% of castration-resistant prostate cancer (CRPC) patients, indicating that AR plays an important role in the development of PCa [35,36]. Significantly higher expression of AR was also observed in balding versus non-balding scalp follicles from men with AGA [13], in line with the present results. In this regard, Hayes et al. [37] described a variant in the AR gene (AR-E211 A allele) associated with a lower risk of metastatic PCa and AGA.

Interleukin 6 (IL-6) is an anti-inflammatory myokine that has been found to activate AR-mediated gene expression via a signal transducer and activator of transcription 3 (Stat3) activator pathway in prostate carcinoma cells [38,39]. IL-6 is also known to increase intracrine androgen levels by enhancing the expression of genes that mediate androgen metabolism in PCa cells [40]. In agreement with the present results, IL-6 was found to be upregulated in balding versus non-balding DP cells and to be produced in response to DHT, suggesting that IL-6 inhibits hair growth as a paracrine mediator from the DP [41]. In addition, some authors have proposed a significant role for IL-6 in the development of AGA and the malignant progression of PCa and have described the effects on PCa and AGA of anti-IL6 therapies [42,43].

ETV1 is a member of the E-twenty-six (ETS) family of transcription factors, which regulate many target genes that modulate biological processes (e.g., cell growth, proliferation, differentiation, and apoptosis) through their activation or repression [44]. ETV1 upregulates the expression of AR target genes and promotes autonomous testosterone production, and its overexpression is associated with aggressive PCa [45]. A recent study also reported that ETV1 can activate the *TWIST1* promoter, which regulates PCa cell invasion [46]. To date, no scientific evidence has been published correlating ETV1 overexpression with AGA, although Chew et al. [47] found that *TWIST1* is upregulated in DP cells from balding versus non-balding scalps. Hence, ETV1 overexpression in AGA could activate the *TWIST1* promoter, which would, in turn, upregulate AR expression [48].

Adenomatous polyposis coli (*APC*) is a key tumor suppressor gene. Its mutation has been detected in numerous cancers, including PCa, and its loss has been correlated with reduced overall survival in cancer patients [49]. APC is a member of the Wnt signaling pathway and a critical component of the β-catenin destruction machinery [50]. Wnt/β-catenin signaling also plays a pivotal role in embryonic HF morphogenesis, development, and regeneration during the hair cycle [51,52]. It has been reported that genetic or pharmacologic ablation of β-catenin could cause the failure of HF formation, indicating the importance of the Wnt signal in hair development [53]. Wnt signaling is regulated by the presence/absence of β-catenin. Thus, the signal transduction pathway is OFF when the Wnt signal is absent because β-catenin is rapidly destroyed; however, β-catenin accumulates in the cytoplasm when the signal is present and is transported to the nucleus, where it activates transcriptional programs involved in cell proliferation in the hair matrix and DP [54]. Furthermore, crosstalk between androgen and Wnt/β-catenin signaling has been attributed to a major role in AGA pathogenesis, given that DHT was found to inhibit the canonical Wnt signaling pathway [55]. The increased APC observed in HFs from the present series of men with AGA could alter the regulation of HF development, given its role as a negative regulator of the Wnt/β-catenin pathway.

FOXO1 is a member of the FOXOs family, which is involved in cell proliferation, differentiation, and apoptosis through the regulation of multiple genes [56]. FOXO1 is considered a key tumor suppressor in cancer, including PCa [56], and was found to be markedly downregulated in PCa samples [57]. To date, there is no scientific evidence of a correlation between FOXO1 and AGA; however, FOXO1 plays a critical role in qualitative control of the VEGF signaling pathway [58], and VEGF promotes perifollicular vascularization, hair growth rates, and increased follicle and hair size [59]. Consequently, decreased FOXO1 in HF could stimulate the development of AGA.

Glutathione peroxidase 3 (GPX3) is a glutathione peroxidase and plays a critical role in the detoxification of free radicals, thereby protecting cells from oxidative stress. GPX3 functions as a tumor suppressor, and its downregulation is widely observed in PCa [60]. In relation to AGA, oxidative stress in DP cells can override innate antioxidant defense mechanisms and produce cell apoptosis in HFs [61]. Moreover, excessive reactive oxygen species (ROS) can trigger the premature senescence of DP cells and suppress the telogen-to-anagen conversion of HFs via androgen signaling [62]. In this way, decreased GPX3 mRNA in HFs could increase oxidative stress and induce HF dysregulation, causing AGA.

## 4. Conclusions

These results confirm the involvement of 5α-R isozymes at the molecular level in the etiology of AGA in young men, who showed a significant increase in the expression of 5α-R2. Importantly, 5α-R isozymes did not show the same pattern of expression in all of these men, and their quantification with a low-invasive methodology may facilitate early selection of the appropriate 5α-R inhibitor treatment. The observation of alterations in some PCa-related genes may open the way to novel therapeutic approaches to AGA.

## 5. Materials and Methods

### 5.1. Subjects and Sampling

This study included 10 men aged 20–30 years diagnosed with grade II alopecia according to the Ludwig classification and 10 healthy men (controls) with no hair loss or thinning. Individuals with alopecia other than AGA (e.g., hair loss from autoimmune disease) were excluded. Neither AGA patients nor control cohorts were under pharmacological treatment for alopecia or any other disease. Informed consent was obtained from all participants before the study.

In all participants, human hair samples were obtained with a trichogram from the vertex area of the scalp, using tightly closing epilation forceps to pluck 30–40 hairs. Differences in tractional (epilation) force between the inner and outer areas of the sample were minimized because all samples were taken in identical conditions by the same dermatologist, following the protocol described by Serrano-Falcon et al. [63]. Epilated hair roots were placed on a glass slide and covered with a coverslip for a light microscopy study of hair root types. The proximal end of the hair shaft was examined to determine the anagen, telogen, or catagen phase. At root examination, the anagen/telogen ratio was about 80/20. Only hair roots in the anagen phase were selected for study. Anagen hair shafts are longer, with a uniform diameter, a rectangular shape, and a slight distal angle. Anagen hair bulbs are darkly pigmented triangular or delta-shaped bulbs at an angle to the hair shaft with the presence of an inner root sheath. Samples from patients contained hair longer than 2 cm, with some miniaturized hair but no fuzz. For each patient, the 10 anagen hairs with the most intact sheaths were selected for this study. Samples were immediately transferred to individual Eppendorf tubes prefilled with 0.5 mL RNAlater (Life Technologies, Waltham, MA, USA) to avoid RNA degradation and were stored at room temperature for 24 h, followed by storage at −80 °C.

### 5.2. RNA Isolation

Around 10 hair roots in anagen phase per participant were homogenized in phenol–guanidine isothiocyanate (Trizol) (Life Technologies, Waltham, MA, USA) following Sanger Institute© instructions. RNA samples were then treated with Turbo DNase (Thermo Fisher Scientific, Waltham, MA, USA) to remove contamination with genomic DNA. A NanoDrop 1000 spectrophotometer (Thermo Fisher Scientific, Waltham, MA, USA) was used to determine the concentration and purity of the total RNA extracted, measuring the OD260 and OD260/280 ratios, respectively, in RNase-free H_2_O. Three replicates of each RNA sample were measured, averaging concentrations. The OD260/280 ratio was always between 1.8 and 2.0. Electrophoresis with ethidium bromide staining was carried out to evaluate total RNA integrity. Total RNA samples were stored at −80 °C until analysis.

### 5.3. Reverse Transcription and Quantitative Real-Time PCR

First-strand cDNA was synthesized from 1 µg of total RNA following Castro et al. [64]. Absolute quantification of the mRNA of 5α-R1, 5α-R2, 5α-R3, and aromatase in plucked hairs was performed using real-time RT-PCR using the Techne Quantica™ Real-time PCR system (Burlington, NJ, USA) with SYBR Green PCR Master Mix (Promega, Madison, WI, USA). Standard curves were generated as described by Fronhoffs et al. [65]. The cRNA was purified with Turbo-DNAse (Thermo Fisher Scientific, Waltham, MA, USA), and its purity and concentration were measured spectrophotometrically. The quantity of mRNA was expressed as the number of mRNA copies per microgram of total RNA, and the cRNA standard was serially diluted from 1 × 10^2^ to 1 × 10^9^ copies/μL. The PCR profile was as follows: denaturation at 94 °C for 30 s, annealing at 55 °C for the SRD5A1 gene, 54 °C for the SRD5A2 gene, 60 °C for the SRD5A3 gene, 60 °C for the CYP19A1 gene for 30 s, and extension at 72 °C for 30 s. The number of cycles was always 40. At the end of the amplification phase, melting curve analysis was performed on the products formed to confirm that a single PCR product was detected with the SYBR Green dye. All reactions were run in triplicate, and no cDNA was added to negative reactions.

Primers for 5α-R1 (SRD5A1 mRNA, Genbank accession no. NM_001047.3), 5α-R2 (SRD5A2 mRNA, GenBank accession no. NM_000348.3), 5α-R3 (SRD5A3 mRNA, GenBank accession no. NM_024592.4), and aromatase (CYP19A1 mRNA, GenBank accession no. NM_000103.3) were designed using Primer3Plus software v3.3.0. Primer sequences (5′–3′) are given in Table 3.

### 5.4. Human PCa PCR Array

A pathway-specific PCR array (Human PCa RT2 Profiler PCR Array, Qiagen, Hilden, Germany) was used to analyze the expression (in hair roots from men with AGA and controls) of 84 genes implicated in PCa, 5 housekeeping genes, and 3 control genes (genomic DNA control, reverse transcription controls, and positive PCR controls). This array covers genes involved in ARs, PI3 kinase/AKT, PTEN signaling, cell cycle, apoptotic pathways, and genes reported to have differentially methylated promoters in PCa (Table 2). The RT2 First Strand Kit (Qiagen, Hilden, Germany) was used to synthesize first-strand cDNA from 1 μg of total RNA, following the manufacturer’s instructions. The RT2 Profiler PCR Array (Qiagen, Hilden, Germany, Cat. no. PARN 135 ZA) was applied with RT2 SYBR Green qPCR Mastermix (Qiagen, Hilden, Germany, Cat. no. 330401) to analyze the cDNA, following the manufacturer’s instructions. Briefly, 102 μL of cDNA was mixed with 2 × RT2 SYBR Green Mastermix and RNase-free H_2_O up to a total volume of 2.700 μL, and 25 μL of the PCR component mix was then placed in each well of the PCR array. Quantitative real-time PCR reactions were performed with a QuantStudio 3 Real-time PCR system (Thermo Fisher Scientific, Waltham, MA, USA), heating samples at 95 °C for 10 min, followed by 40 cycles of denaturation at 95 °C for 15 s, and then annealing and elongation at 60 °C for 1 min. Normalization of Ct values was based on automatic selection from the full panel of reference genes (*HPRT1*, *RPLP0*). Quality control was confirmed using the RT2 Profiler PCR Array Data Analysis Software v3.5 (Qiagen, Hilden, Germany). Only results that passed quality tests for PCR array reproducibility and genomic DNA contamination were included. Three independent PCR array assays were performed for each group. Each group comprised a pool of hair root samples from different individuals.

### 5.5. Statistical Analysis

The non-parametric Mann–Whitney U test was used for comparisons because the data distribution was found to be non-normal using the Kolmogorov–Smirnov test. GraphPad Prism 5.0 software (San Diego, CA, USA) was used for the statistical analysis. *p* < 0.05 was considered significant. PCR array data were analyzed using the web-based software provided by the manufacturer (https://gene globe.qiagen.com/es/analyze, accessed on 14 July 2022), which uses threshold cycle (Ct) values and offers automatic quantification using Student’s t-statistics and the classical ΔΔCt method [66]. Briefly, ΔCt values were calculated by normalizing the average Ct values for each gene to the expression of housekeeping genes. The software permits the definition of the best reference genes for normalization. Ct values above 35 were excluded from data analyses. The ΔΔCt values were obtained via normalization to the mean expression of each gene in controls. Fold-change values were based on comparisons between triplicate 2^(−ΔΔCt)^ values for each gene in the AGA group and those in the control group. The false discovery rate method of Benjamini and Hochberg [67] was used to correct for multiple testing. *p* < 0.05 was considered statistically significant.

## Figures and Tables

**Table 1:** List of PCR array genes significantly altered in young men with AGA. Fold-Change (2^(−DDCt)^) is the normalized gene expression in the test sample divided by the normalized gene expression in the control sample. *p*-values are calculated based on a Student’s *t*-test of the replicate 2^(−DCt)^ values for each gene in the control group versus the young men with AGA.

Gene Symbol	Description	Fold Changes	*p*
*APC*	Adenomatous polyposis coli	3.62	0.001070
*AR*	Androgen receptor	4.87	0.047702
*ETV1*	Ets variant 1	2.18	0.028468
*IL6*	Interleukin 6	4.35	0.019576
*FOXO1*	Forkhead box O1	−4.00	0.009073
*GPX3*	Glutathione peroxidase 3	−2.56	0.035205

**Table 2:** Functional gene grouping.

Differentially Methylated Promoters	*APC, AR, CAV1, CCNA1, CDH1 (*E-Cadherin*), CDKN2A (*P16INK4A*), DKK3, DLC1, EDNRB, GPX3, GSTP1, MGMT (*AGT*), MSX1, PDLIM4 (*RIL*), PTGS2 (*COX2*), RARB, RASSF1, SFRP1, SLC5A8, TIMP2, TNFRSF10D, ZNF185.*
Upregulated in Prostate Cancer	*ARNTL(*BMAL1*), CAMSAP1, DDX11, ECT2, ETV1, HAL, IGFBP5, KLK3, MTO1, PDPK1, RBM39, SOCS3, SOX4, SUPT7L.*
Downregulated in Prostate Cancer	*CCND2, CLN3, GCA, IGF1, LGALS4, LOXL1, PPP2R1B, SFRP1, SLC5A8, TFPI2, USP5.*
Metastatic Potential	*CREB1, KLHL13, MAX, NDRG3, PES1, SCAF11, SEPT7.*
Androgen Receptor Signaling	*AR, CAV1, CCND1, DAXX, EGFR(*ERBB1*), FOXO1, GNRH1, IGF1, IL6, NFKB1, NRIP1, PTEN, SHBG, TGFB1I1, TIMP2, TIMP3, VEGFA.*
AKT & PI3 Kinase Signaling	*AKT1, AR, BCL2, CCND1, CCND2, CDH1 (*E-Cadherin*), CDKN2A (*P16INK4A*), EGFR(*ERBB1*), FOXO1, GNRH1, IGF1, IL6, MAPK1 (*ERK2*), NFKB1, PDPK1, PTEN, TIMP2, TIMP3, TNFRSF10D, TP53 (*p53*), VEGFA.*
PTEN Signaling	*AKT1, EGFR (*ERBB1*), GNRH1, IGF1, IL6, MAPK1 (*ERK2*), PDPK1, PTEN, TIMP2, TIMP3, TP53 (*p53*), VEGFA.*
Apoptosis	*BCL2, CASP3, CDKN2A (*P16INK4A*), EGFR (*ERBB1*), ETV1, GNRH1, IGF1, IL6, MAPK1 (*ERK2*), NFKB1, PTEN, TIMP2, TIMP3, TP53 (*p53*), VEGFA.*
Cell Cycle	*APC, BCL2, CASP3, CAV2, CCNA1, CCND1, CCND2, CDKN2A (*P16INK4A*), EGFR (*ERBB1*), IGF1, PPP2R1B, PTEN, PTGS1 (*COX1*), PTGS2 (*COX2*), TP53 (*p53*).*
Transcription Factors	*AR, ARNTL (*BMAL1*), CDKN2A (*P16INK4A*), CREB1, DAXX, EGR3, ERG, ETV1, FOXO1, MAX, MSX1, NFKB1, NKX3-1, NRIP1, RARB, RBM39, SOX4, SREBF1, SUPT7L, TP53 (*p53*).*
Fatty Acid Metabolism	*ACACA, CAMKK1, FASN, HMGCR, IGF1, PRKAB1, SREBF1, STK11 (*LKB1*).*
Other Prostate Cancer Genes	*MKI67, TMPRSS2.*

**Table 3:** Primer sequences (5′–3′) and PCR conditions for RT-PCR analysis.

Primer	Forward	Reverse	Size	Annealing Tª (°C)
SRD5A1	AGCCATTGTGCAGTGTATGC	AGCCTCCCCTTGGTATTTTG	136	55
SRD5A2	TGAATACCCTGATGGGTGG	CAAGCCACCTTGTGGAATC	154	54
SRD5A3	TCCTTCTTTGCCCAAACATC	CTGATGCTCTCCCTTTACGC	212	60
CYP19A1	TATTAGGGCCCTGTGTCTGC	TGGGTTGGGACTTTTCCTCC	193	60

## Data Availability

All data generated or analyzed during this study are included in this article. Further inquiries can be directed to the corresponding author.
